# Attitudes, skills and implementation of evidence-based practice: a national cross-sectional survey of licensed naprapaths in Sweden

**DOI:** 10.1186/s12998-023-00473-5

**Published:** 2023-01-20

**Authors:** Tobias Sundberg, Matthew J. Leach, Stina Lilje, Oliver P. Thomson, Gary Fryer, Per J. Palmgren, Jon Adams, Eva Skillgate

**Affiliations:** 1 Musculoskeletal and Sports Injury Epidemiology Center, Department of Health Promotion Science, Sophiahemmet University, Stockholm, Sweden; 2 National Centre for Naturopathic Medicine, Southern Cross University, East Lismore, NSW Australia; 3 Australian Research Centre in Complementary and Integrative Medicine, School of Public Health, Faculty of Health, University of Technology Sydney, Sydney, NSW Australia; 4 Musculoskeletal and Sports Injury Epidemiology Center, Unit of Intervention and Implementation Research on Worker Health, Institute of Environmental Medicine, Karolinska Institutet, Stockholm, Sweden; 5 Research Centre, University College of Osteopathy, London, UK; 6 College of Health and Biomedicine, Victoria University, Melbourne, Vic Australia; 7 Department of Learning, Informatics, Management and Ethics, Karolinska Institutet, Stockholm, Sweden

**Keywords:** Evidence-based practice, Naprapathy, Health care surveys, Cross-sectional studies, Implementation science

## Abstract

**Background:**

Evidence-based practice (EBP) is fundamental to the delivery of high-quality, safe and effective health care. Naprapaths, manual therapy providers that specialize in the treatment of musculoskeletal pain and dysfunction, became a Swedish licensed health profession in 1994. This study investigated the attitudes, skills and implementation of EBP among licensed naprapaths in Sweden.

**Methods:**

Licensed naprapaths (n = 950) of Svenska Naprapatförbundet (the Swedish Naprapathy Association) were invited by email to take part in this cross-sectional anonymous online study using the Evidence-Based Practice Attitude and Utilisation Survey in February 2019.

**Results:**

Fourteen percent (137/950) of invited naprapaths completed the survey. There was an approximately equal gender divide among responders; most were in the mid-career age range, practiced in city areas, and had a university or college certificate/diploma as their highest qualification. The majority of naprapaths agreed or strongly agreed that EBP was necessary in the practice of naprapathy, assisted them in making care decisions, and improved the quality of patient care. Naprapaths’ self-reported skills in EBP were mostly in the moderate to moderate-high range. The majority of participating naprapaths reported infrequent implementation of EBP. Perceived minor or moderate barriers to EBP uptake included a lack of colleague support for EBP and a lack of relevant resources. Access to the internet and free online databases were reported as very useful enablers to improving EBP uptake.

**Conclusions:**

The licensed naprapaths participating in this survey reported positive attitudes toward EBP, moderate levels of EBP skills, and infrequent implementation of EBP.

## Background

Evidence-based practice (EBP)—the synthesis of the best available research evidence with clinical expertise and patient preferences [1]—is fundamental to the delivery of high-quality, safe and effective health care. The principles and application of EBP have been embraced widely across healthcare fields, including musculoskeletal health professionals such as physiotherapists [2], osteopaths [3], and chiropractors [4]. Although the nature of what constitutes best evidence has been debated [5–7], a ‘renaissance’ of EBP has been promoted by re-positioning the individual patient at the centre of evidence-based decision-making, enhancing the usability of evidence and more critically incorporating professional judgement and the patient’s personal context [8, 9]. While this resurgence of EBP has occurred mainly in the medical profession, similar discussions have taken place in the physical and manual therapy professions [10, 11]. Previous studies of chiropractors in the US, Canada and Sweden [4, 12, 13], and among osteopaths in the UK, Spain, Italy, Sweden and Australia [13–17], suggest that while these manual therapy professions infrequently implement EBP, they do overall hold positive attitudes toward EBP and possess moderate skills in EBP.

Naprapathy, a manual therapy profession founded by US chiropractor Oakley Smith in 1905, shares many characteristics with the chiropractic profession, such as the reliance on manual therapy techniques to diagnose and treat musculoskeletal pain and associated disability [18]. However, in contrast to chiropractic beliefs at the time, naprapathy was against the theory of vertebral 'subluxations' as a basis of disease and instead considered soft and connective tissues as primary causes of pain and dysfunction [18]. Contemporary licensed naprapaths in Sweden specialize in the treatment of patients with musculoskeletal pain and dysfunction, typically by using manual therapy techniques including massage, stretching, trigger point therapy, joint mobilizations and manipulations, combined with advice on home exercises and staying active [18].

In Sweden, naprapathy has been a licensed health profession since 1994 [19, 20] with its practice regulated by the Swedish National Board of Health and Welfare [21]. In 2019, there were almost twice the number of licensed naprapaths (n = 1320) to licensed chiropractors (n = 769) working in Sweden [22]. Despite these circumstances, the integration of naprapathy into conventional public funded health care services and settings in Sweden remains scarce. The training of naprapaths in Sweden is currently only available at one private college Naprapathögskolan (The Scandinavian College of Naprapathic Manual Medicine), which is outside of the public-funded Swedish higher education system where conventional health professions such as physiotherapists, physicians, nurses, and psychologists receive training. As naprapathy is a health profession licensed by the Swedish National Board of Health and Welfare, which is a significant recognition of quality assurance for the public and other health care providers, naprapaths must adhere to the core principles of EBP [23]. However, to date there has been no empirical investigation of EBP in naprapathy.

In direct response to this knowledge gap we aimed to investigate the attitudes, skills and implementation of EBP among Swedish licensed naprapaths. Specifically, we endeavoured to answer the following research questions:

1. What is the attitude toward EBP among Swedish licensed naprapaths?
2. How do Swedish licensed naprapaths rate their level of skill in EBP?
3. To what extent do Swedish licensed naprapaths implement EBP activities?
4. What factors do Swedish licensed naprapaths identify as barriers to, and enablers of EBP uptake?
5. What is the association between the demographics of Swedish licensed naprapaths and their attitudes, skills and implementation of EBP?

## Methods

### Design and setting

A national cross-sectional anonymous online survey of licensed naprapaths in Sweden.

### Sample

The survey was accessible to licensed naprapaths of Svenska Naprapatförbundet (the Swedish Naprapathy Association), which had a target population of 950 licensed naprapaths as of February 2019 (Swedish Naprapathy Association 2019). Based on this target population, a response distribution of 50%, margin of error of 8%, and a confidence interval of 95% for any item in the survey, we needed to survey at least 130 naprapaths [24].

### Measurement

Eligible participants were invited to complete the 84-item Evidence-Based practice Attitude and utilization SurvEy (EBASE). This multidimensional, self-administered questionnaire addresses six domains related to EBP: attitude (Part A), skill-level (Part B), training and education (Part C), utilisation (Part D), barriers (Part E), and enablers (Part F). Participant demographic information is collected in Part G of the questionnaire. Three subscores can be generated from EBASE, including: (i) attitude subscore (with scores ranging from 8 [predominantly strongly disagree] to 40 [predominantly strongly agree]); (ii) skill subscore (with scores ranging from 13 [primarily low-level skill] to 65 [primarily high-level skill]); and (iii) use subscore (with scores ranging from 0 [mainly infrequent use] to 24 [mainly frequent use]).

EBASE has undergone psychometric evaluation, and has demonstrated good internal consistency, content and construct validity, and acceptable test–retest reliability [25, 26]. Further, the survey is well-established and has been administered to diverse groups of health care professionals across multiple countries [4, 12, 16, 17, 27–30].

The survey was translated into Swedish by adapting the WHO process for translating instruments [31]. The survey was first translated from English into Swedish. An external translator then contributed to backwards translation, and cognitive interviewing was conducted with a survey developer. The Swedish version of EBASE was pilot tested using a convenience sample of ten respondents representing various professional backgrounds including naprapathy, physiotherapy, chiropractic, osteopathy, social work, and administration. Some survey items underwent minor modification to ensure suitability for the naprapathy target population; for example, ‘Australian States’ was replaced with ‘Counties of Sweden’, and treatments typically provided in an initial consultation were more closely aligned with Swedish naprapathic practice. These modifications did not alter the meaning of the items.

### Recruitment and data collection

The Swedish Naprapathy Association emailed a link to the anonymous survey to their licensed naprapath members (n = 950) in February of 2019. The survey was open for two months, during which time the invitees received two reminders to participate at one and three weeks after the first invite. The survey was administered via the secure web-based survey hosting system Sunet Survey, which is widely used in Swedish higher education [32].

### Data analysis

Survey data were imported into IBM® SPSS® Statistics 25.0 (Armonk, New York, IBM Corp) for coding and analysis. Omitted responses were reported as missing data. Frequencies and percentages were used to describe categorical data. Numerical data were treated as ordinal and non-parametric, for which medians were used as measures of location and the interquartile range as measures of dispersion. We used Cramer’s V to examine the relationships between nominal-level variables (e.g. gender, geographical region), and Kendall’s Tau correlation coefficient (Ƭ) to test for associations between ordinal-level variables (e.g. age, highest qualification). Coefficients ranging between 0.10 and 0.29 were interpreted as a weak correlation, 0.30–0.49 a moderate correlation, and 0.50–1.00 a strong correlation [33].

## Results

One hundred and thirty-seven licensed naprapaths completed the survey yielding a response rate of 14.4% (137/950).

### Sample characteristics

There was an approximately equal gender divide (52.6% men) among participating naprapaths. Most participants were aged between 30 and 49 years (62.8%), and resided in a city location (75.9%) (Table 1). Two-thirds (65.0%) had reported a university or college certificate/diploma as their highest qualification, with 58.4% obtaining this qualification ≥ 11 years prior to the survey. Accordingly, most respondents had practiced in the field of naprapathy for 11 or more years (61.3%), with most working 31 or more hours per week in clinical practice (65%). Few respondents reported participating in research (17.5%; ≥ 1 h/week) or teaching higher education (13.1%; ≥ 1 h/week). Treatments/management typically provided in the first naprapathic consultation were diverse, with joint manipulation (91.2%), exercise and physical activity advice or instruction (85.4%), home exercise and activities of daily living advice or instruction (84.7%), trigger point therapy (84.7%) and massage/soft-tissue mobilization (81.8%) among the top five most frequently reported interventions.

**Table 1:** Demographic characteristics of sample (n = 137)

Characteristic	Frequency (%)
*Age*	
20–29 years	12 (8.8)
30–39 years	50 (36.5)
40–49 years	36 (26.3)
50–59 years	32 (23.4)
60–69 years	6 (4.4)
70 + years	0 (0)
Missing	1 (0.7)
*Gender*	
Woman	61 (44.5)
Man	72 (52.6)
Other	1 (0.7)
Do not wish to state	2 (1.5)
Missing	1 (0.7)
*Highest qualification*	
High school certificate	4 (2.9)
Vocational Degree/Diploma	10 (7.3)
University or College Certificate/Diploma	89 (65.0)
Bachelor degree	4 (2.9)
Master’s degree (1 year)	5 (3.6)
Master’s degree (2 years)	5 (3.6)
PhD/Doctorate	2 (1.5)
Other	17 (12.4)
Missing	1 (0.7)
*Years since receiving highest qualification*	
< 1 year	2 (1.5)
1–5 years	27 (19.7)
6–10 years	27 (19.7)
11–15 years	26 (19.0)
16 + years	54 (39.4)
Missing	1 (0.7)
*Years practiced in the field of naprapathy*	
< 1 year	1 (0.7)
1–5 years	23 (16.8)
6–10 years	27 (19.7)
11–15 years	25(18.2)
16 + years	59 (43.1)
Missing	2 (1.5)
*Hours per week in clinical (naprapathic) practice*	
0 h	3 (2.2)
1–15 h	13 (9.5)
16–30 h	31 (22.6)
31–45 h	80 (58.4)
46 + hours	9 (6.6)
Missing	1 (0.7)
*Hours per week participating in research*	
0 h	112 (81.8)
1–15 h	22 (16.1)
16–30 h	2 (1.5)
31–45 h	0 (0.0)
46 + hours	0 (0.0)
Missing	1 (0.7)
*Hours per week teaching higher education*	
0 h	118 (86.1)
1–15 h	15 (10.9)
16–30 h	2 (1.5)
31–45 h	1 (0.7)
46 + hours	0 (0.0)
Missing	1 (0.7)
Treatments/management typically provided in initial naprapathic consultation*	
Joint manipulation (e.g. HVLA)	125 (91.2)
Exercise and physical activity advice or instruction	117 (85.4)
Home exercise and ADL advice or instruction	116 (84.7)
Trigger point therapy	116 (84.7)
Massage/soft-tissue mobilization	112 (81.8)
Joint mobilisation	108 (78.8)
Stretching	97 (70.8)
Physical exercise/rehabilitation training	95 (69.3)
Ergonomic advice or instruction	85 (62.0)
Health/lifestyle advice or instruction	75 (54.7)
Acupuncture/acupressure/dry needling	69 (50.4)
Traction	61 (44.5)
Referral to other healthcare provider	60 (43.8)
Non-prescription pharmaceutical advice or instruction	46 (33.6)
Taping	44 (32.1)
TENS	38 (27.7)
Referral to other health service	36 (26.3)
Dietary advice or instruction	31 (22.6)
Laser therapy	28 (20.4)
Shockwave therapy	24 (17.5)
Heat/cold treatment	16 (11.7)
Nutritional supplementation advice	16 (11.7)
Ultrasound	11 (8.0)
Other	8 (5.8)
*Clinical setting in which naprapathy is predominantly practiced*	
With a group of naprapaths	52 (38.0)
Solo practice	31 (22.6)
With CAM & conventional providers	21 (15.3)
With a group of conventional providers	17 (12.4)
Other	8 (5.8)
Within an educational institution (e.g. university)	4 (2.9)
With a group of CAM providers	3 (2.2)
Missing	1 (0.7)
*County of Sweden*	
Stockholm	64 (46.7)
Västra Götaland	15 (10.9)
Skåne	14 (10.2)
Västernorrland	8 (5.8)
Halland	5 (3.6)
Uppsala	5 (3.6)
Other (< 5 respondends per county: Blekinge, Östergötland, Norrbotten, Gävleborg, Jämtland, Södermanland, Örebro, Dalarna, Värmland, Västerbotten, Västmanland)	23 (16.8)
Missing	3 (2.2)
*Geographical region*	
City (Central business district)	104 (75.9)
Suburbs	31 (22.6)
Rural/remote region	0 (0.0)
Missing	2 (1.5)

*ADL* activities of daily living; *CAM* complementary and alternative medicine; *HVLA* high-velocity low amplitude; *TENS* transcutaneous electrical nerve stimulation

*Multiple response question

### Attitudes toward EBP

Respondents reported a largely favourable attitude toward EBP, as evidenced by a median attitude subscore of 31 (IQR 28,34; range 15–37; with scores ranging between 24.1 and 31.9 reflecting a predominantly neutral to agree response). Most respondents agreed or strongly agreed that professional literature and research findings were useful in their day-to-day practice (94.2%), EBP was necessary in the practice of naprapathy (93.4%), and that they were interested in learning or improving the skills necessary to incorporate EBP into their practice (89.8%) (Table 2). The majority of respondents disagreed or strongly disagreed there was a lack of evidence from clinical trials to support the treatments in their practice (66.4%), or that the adoption of EBP placed an unreasonable demand on their practice (75.2%).

**Table 2:** Respondent attitudes toward evidence-based practice (n = 137)

	1 Strongly Disagree n (%)	2 Disagree n (%)	3 Neutral n (%)	4 Agree n (%)	5 Strongly Agree n (%)	Median (IQR)
EBP is necessary in the practice of naprapathy	1 (0.7)	2 (1.5)	6 (4.4)	54 (39.4)	**74 (54.0)**	5 (4,5)
Professional literature (i.e. journals & textbooks) and research findings are useful in my day-to-day practice	0 (0.0)	1 (0.7)	7 (5.1)	58 (42.3)	**71 (51.8)**	5 (4,5)
I am interested in learning or improving the skills necessary to incorporate EBP into my practice	2 (1.5)	2 (1.5)	10 (7.3)	54 (39.4)	**69 (50.4)**	5 (4,5)
EBP improves the quality of my patient’s care	3 (2.2)	3 (2.2)	11 (8.0)	**61 (44.5)**	59 (43.1)	4 (4,5)
EBP assists me in making decisions about patient care	1 (0.7)	4 (2.9)	14 (10.2)	**59 (43.1)**	**59 (43.1)**	4 (4,5)
Prioritizing EBP within naprapathic practice is fundamental to the advancement of the profession	5 (3.6)	9 (6.6)	18 (13.1)	**61 (44.5)**	44 (32.1)	4 (4,5)
EBP takes into account my clinical experience when making clinical decisions	4 (2.9)	17 (12.4)	26 (19.0)	**59 (43.1)**	31 (22.6)	4 (3,4)
EBP takes into account a patient’s preference for treatment	13 (9.5)	**43 (31.4)**	30 (21.9)	41 (29.9)	10 (7.3)	3 (2,4)
There is a lack of evidence from clinical trials to support most of the treatments I use in my practice	28 (20.4)	**63 (46.0)**	22 (16.1)	18 (13.1)	6 (4.4)	2 (2,3)
The adoption of EBP places an unreasonable demand on my practice	26 (19.0)	**77 (56.2)**	21 (15.3)	9 (6.6)	4 (2.9)	2 (2,3)

*EBP* evidence-based practice; *IQR* interquartile range; main response in bold

#### Demographical association

Attitude subscore (categorised by quartiles) was positively associated with hours per week participating in research (Ƭ = 0.209, p = 0.006), albeit weakly. There were no statistically significant associations between attitude subscore and other demographic factors.

### Skills in EBP

Respondents reported a median skill subscore of 39 (IQR 32,46; range 16–65), which was indicative of a mostly moderate level of perceived skill in EBP (with scores ranging between 26.1 and 39.0 suggestive of a predominantly low-moderate to moderate skill level). More than three-quarters of respondents reported a moderate to moderate-high level of skill in clinical problem identification (i.e. identifying answerable clinical questions [79.6%] and identifying knowledge gaps in practice [76.6%]) (Table 3). Eleven of the 13 skills were largely perceived to be in the moderate to moderate-high range. The lowest levels of perceived skill related to the conduct of systematic reviews (67.2% in the low to low-moderate range) and clinical research (69.3% in the low to low-moderate range).

**Table 3:** Participants' perceived skill level in evidence-based practice (n = 137)

	1 Low n (%)	2 Low-moderate n (%)	3 Moderate n (%)	4 Moderate-high n (%)	5 High n (%)	Median (IQR)
Identifying answerable clinical questions	1 (0.7)	4 (2.9)	50 (36.5)	**59 (43.1)**	23 (16.8)	4 (3,4)
Identifying knowledge gaps in practice	2 (1.5)	7 (5.1)	45 (32.8)	**60 (43.8)**	23 (16.8)	4 (3,4)
Locating professional literature	5 (3.6)	24 (17.5)	39 (28.5)	**41 (29.9)**	28 (20.4)	4 (3,4)
Online database searching	18 (13.1)	29 (21.2)	33 (24.1)	**39 (28.5)**	18 (13.1)	3 (2,4)
Retrieving evidence	17 (12.4)	32 (23.4)	**41 (29.9)**	31 (22.6)	16 (11.7)	3 (2,4)
Critical appraisal of evidence	11 (8.0)	29 (21.2)	**40 (29.2)**	38 (27.7)	19 (13.9)	3 (2,4)
Synthesis of research evidence	16 (11.7)	27 (19.7)	**56 (40.9)**	26 (19.0)	12 (8.8)	3 (2,4)
Applying research evidence to patient cases	5 (3.6)	20 (14.6)	42 (30.7)	**52 (38.0)**	18 (13.1)	4 (3,4)
Sharing evidence with colleagues	13 (9.5)	30 (21.9)	**48 (35.0)**	31 (22.6)	15 (10.9)	2 (3,4)
Using findings from clinical research	7 (5.1)	14 (10.2)	**51 (37.2)**	49 (35.8)	16 (11.7)	3 (3,4)
Using findings from systematic reviews	20 (14.6)	34 (24.8)	**36 (26.3)**	31 (22.6)	16 (11.7)	3 (2,4)
Conducting systematic reviews	45 (32.8)	**47 (34.3)**	21 (15.3)	19 (13.9)	5 (3.6)	2 (1,3)
Conducting clinical research	**54 (39.4)**	41 (29.9)	25 (18.2)	11 (8.0)	6 (4.4)	2 (1,3)

*IQR* Interquartile range; main response in bold

#### Demographical association

A positive association was evident between skill subscore (categorised by quartiles) and hours per week participating in research (Ƭ = 0.389, p < 0.001), hours per week teaching higher education (Ƭ = 0.216, p = 0.012), and gender with male participants reporting higher skill levels (V = 0.207, p = 0.042). Years since receiving the highest qualification (Ƭ = − 0.254, p < 0.001) and years practicing as a naprapath (Ƭ = -0.152, p = 0.037) were shown to be inversely associated with skill subscore. There were no statistically significant associations between skill subscore and other demographic variables.

### Implementation of EBP

Respondents engaged in EBP activities mostly in the range of 1–10 times/month, reflected by a median use subscore of 8 (IQR 5,17; range 0–24; with scores ranging between 6.1 and 12.0 indicative of use in the 1–10 times/month range). However, the majority of respondents had engaged in most activities no more than 5 times in the previous month (e.g. used an online database [65.7%], read/reviewed clinical research findings [61.3%], used professional literature or research findings to change clinical practice [66.4%], read/reviewed professional literature related to their practice [61.3%]) (Table 4). Interestingly, most respondents indicated that much of their practice was based on clinical research evidence; specifically, 51–75% of practice (40.1% of respondents), 76–99% of practice (31.4%) and 100% of practice (2.2%). Only 10.2% and 16.1% of participants indicated that a very small proportion (1–25%) or small proportion (26–50%) of their practice, respectively, was based on clinical research evidence.

**Table 4:** Participant use of evidence-based practice (i.e. number of times each activity was undertaken within the last month) (n = 137)

	0 0 times n (%)	1 1–5 times n (%)	2 6–10 times n (%)	3 11–15 times n (%)	4 16 + times n (%)	Median (IQR)
I have used an online database to search for practice related literature or research	**48 (35.0)**	42 (30.7)	9 (6.6)	9 (6.6)	29 (21.2)	1 (0,3)
I have read/reviewed clinical research findings related to my practice	36 (26.3)	**48 (35.0)**	15 (10.9)	12 (8.8)	26 (19.0)	1 (0,3)
I have used professional literature or research findings to change my clinical practice	26 (19.0)	**65 (47.4)**	16 (11.7)	4 (2.9)	26 (19.0)	1 (1,2)
I have referred to magazines, layperson / self-help books, or non-government/non-education institution websites to assist my clinical decision-making	30 (21.9)	**57 (41.6)**	13 (9.5)	6 (4.4)	31 (22.6)	1 (1,3)
I have read/reviewed professional literature (i.e. professional journals & textbooks) related to my practice	29 (21.2)	**55 (40.1)**	11 (8.0)	13 (9.5)	29 (21.2)	1 (1,3)
I have consulted a colleague or industry expert to assist my clinical decision-making	14 (10.2)	**56 (40.9)**	19 (13.9)	5 (3.6)	43 (31.4)	1 (1,4)
I have used an online search engine to search for practice related literature or research	16 (11.7)	**50 (36.5)**	20 (14.6)	5 (3.6)	46 (33.6)	2 (1,4)
I have used professional literature or research findings to assist my clinical decision-making	11 (8.0)	**49 (35.8)**	25 (18.2)	8 (5.8)	44 (32.1)	2 (1,4)

*IQR* interquartile range; main response in bold

Respondents used a range of information sources to guide their clinical decision-making (Table 5). The information source used the most (either to a moderate extent or a lot) was traditional knowledge (83.2%). This was followed by consultation with fellow practitioners/experts, personal preference, and textbooks, which were used by 76.6%, 76.6% and 69.3% of respondents, respectively, either to a moderate extent or a lot. Most respondents (83.9%) never used or only used a little experimental/laboratory evidence to inform their clinical decision making (Table 5).

**Table 5:** Information sources used by participants to inform their clinical decision-making (n = 137)

Information source	1 Never used n (%)	2 Used a little n (%)	3 Used to a moderate extent n (%)	4 Used a lot n (%)	5 Always used n (%)	Missing n (%)	Median (IQR)
Traditional knowledge	1 (0.7)	9 (6.6)	45 (32.8)	**69 (50.4)**	13 (9.5)	0 (0.0)	4 (3,4)
Fellow practitioners or experts	1 (0.7)	23 (16.8)	**59 (43.1)**	46 (33.6)	6 (4.4)	2 (1.5)	3 (3,4)
Personal preference	4 (2.9)	22 (16.1)	45 (32.8)	**60 (43.8)**	6 (4.4)	0 (0.0)	3 (3,4)
Textbooks	5 (3.6)	31 (22.6)	**54 (39.4)**	41 (29.9)	5 (3.6)	1 (0.7)	3 (2,4)
Patient preference	5 (3.6)	42 (30.7)	**50 (36.5)**	37 (27.0)	3 (2.2)	0 (0.0)	3 (2,4)
Personal intuition	6 (4.4)	35 (25.5)	**42 (30.7)**	**42 (30.7)**	11 (8.0)	1 (0.7)	3 (2,4)
Published clinical evidence	6 (4.4)	**46 (33.6)**	43 (31.4)	37 (27.0)	5 (3.6)	0 (0.0)	3 (2,4)
Clinical practice guidelines	7 (5.1)	28 (20.4)	**48 (35.0)**	37 (27.0)	13 (9.5)	4 (2.9)	3 (2,4)
Trial and error	8 (5.8)	**57 (41.6)**	42 (30.7)	28 (20.4)	2 (1.5)	0 (0.0)	3 (2,3)
Experimental/laboratory evidence	**72 (52.6)**	43 (31.4)	14 (10.2)	5 (3.6)	1 (0.7)	2 (1.5)	1 (1,2)

*IQR* Interquartile range; main response in bold

#### Demographical association

A weak positive association was found between implementation of EBP by use subscore (categorised by quartiles) and highest qualification (Ƭ = 0.162, p = 0.024), and hours per week participating in research (Ƭ = 0.163, p = 0.033). Use subscore was shown to be inversely associated with years since receiving highest qualification (Ƭ = − 0.159, p < 0.023) and years practicing as a naprapath (Ƭ = -0.182, p = 0.011). There were no statistically significant associations between use subscore and other demographic factors.

### Training in EBP

The majority (52.6%-86.1%) of respondents had completed some degree of training in 5 topics related to EBP. Close to 1 in 3 respondents indicated they had received education during their undergraduate training programme in the areas of evidence-based practice (37.2% of respondents), the application of research evidence to clinical practice (32.8%), critical thinking/analysis (32.2%) and the conduct of clinical research (31.4%). More than one-third of respondents had undertaken no training in conducting clinical research (35%) or conducting systematic reviews or meta-analyses (47.4%).

### Barriers to and enablers of EBP uptake

Thirteen potential barriers to EBP were listed in EBASE. Few (1.5–13.9%) respondents perceived these as ‘major barriers’ to EBP uptake. For five of the listed factors, most (53.3–63.5%) respondents reported these as ‘not a barrier’. The only factors that were largely perceived as ‘minor’ or ‘moderate’ barriers to EBP uptake were lack of colleague support for EBP (65.0%), lack of resources (63.5%), lack of relevance to naprapathic practice (59.9%), lack of industry support for EBP (59.9%) and lack of interest in EBP (53.3%).

From the list of ten potential enablers of EBP uptake, most respondents indicated that these enablers would be moderately to very useful (48.2–95.7%). The factors largely considered by respondents as being very useful in improving EBP uptake were access to the internet in the workplace (86.9%) and access to free online databases in the workplace (54.0%). Over one-half (51.8%) of respondents indicated access to online tools that assist with conducting critical appraisals of research papers would be not or only slightly useful in improving EBP uptake.

## Discussion

This study investigated for the first time, the attitudes, skills and implementation of EBP among Swedish licensed naprapaths. The main findings showed that participants had positive attitudes toward EBP, and moderate levels of EBP skills, whereas their implementation of EBP activities was infrequent. These findings are in line with results from previous studies involving chiropractors [4, 12, 13], and osteopaths [14–17].

### Response rate

Although the response rate (14.4%) for this study was rather low, the required sample size was reached. Furthermore, the response rate surpassed that of other cross-sectional studies using EBASE, including those involving UK osteopaths (7.2%) [17], and Spanish osteopaths (9%) [14]. By contrast, previous Swedish studies using EBASE have yielded relatively higher response rates, including surveys of osteopaths (31%) [34] and chiropractors (33%) [13]. While geographical and disciplinary variations in these populations may be contributing factors, it is also possible that informal collegiate communication among smaller networks of peers in the considerably smaller professional bodies of the Swedish Chiropractic Association (n = 172) [13] and the Swedish Osteopathic Association (n = 249) [34] might have spurred greater interest in study participation than that possible through the larger Swedish Naprapathy Association (n = 950).

### Sample characteristics

There was an approximately equal gender divide among responding naprapaths, with the majority being in the mid-career age range and practicing in city areas. Whether this profile is representative of the broader Swedish naprapathy workforce is unclear due to the lack of accessible data on this profession. Notwithstanding, these responder characteristics are in line with findings from previous investigations of Swedish chiropractors [13] and osteopaths [34]. Most responding naprapaths held a certificate/diploma as their highest qualification, whereas few held a Bachelor's degree or higher. This is to be expected as the one naprapathic college in Sweden, the Scandinavian College of Naprapathic Manual Medicine, is not a part of the state-funded public higher education system, and accordingly, is not granted to award higher academic degrees, such as BSc, MSc or PhD [35]. Nevertheless, at least 1 in 10 naprapaths had pursued academic training and degrees beyond their naprapathic manual therapy training, which may be a relevant characteristic to monitor in future studies to inform the continued professionalization and academisation processes of the Swedish naprapathic profession.

Most naprapaths reported working in clinical practice, commonly practicing alone or together with colleagues of the same or a complementary or conventional health profession. By contrast, few naprapaths reported participating in research or teaching in higher education. These characteristics are consistent across previous EBP studies involving manual therapy professions, both in Europe [14, 17, 34], and in Australia [16]. The most frequent types of naprapathic treatment, reported by more than 80% of respondents, were massage and soft-tissue mobilization, trigger point therapy, joint manipulation, and various forms of exercise and physical activity advice or instruction. The combination of manual therapy and exercise activities is consistent with current clinical practice guideline recommendations for the management of patients with back pain [36], which coincidently is one of the most common ailments treated by naprapaths in Sweden [37].

### Attitudes toward EBP

Participating naprapaths reported generally favourable attitudes toward EBP, which is similar to the findings of previous studies involving osteopaths and chiropractors [4, 13–17, 34]. The vast majority (93.4%) of participants in our study agreed or strongly agreed that EBP was necessary in the practice of naprapathy. This represents a stronger level of agreement for the role of EBP in practice than that reported by US chiropractors (85.3%) [4], Australian osteopaths (84.6%) [16], UK osteopaths (76.5%) [17], and Swedish osteopaths (80.8%) [34]. By contrast, the level of agreement was not as high as that reported by Swedish chiropractors (98.2%) [13].

Encouragingly, most naprapaths in our study were interested in developing skills necessary to implementing EBP. Despite this level of interest, and the strong level of support for the role of EBP in naprapathy, approximately forty percent of naprapaths disagreed or strongly disagreed that EBP takes into account the patient’s preference for treatment. Given that patient preference is a key feature in the definition of evidence-based medicine [1], this finding suggests that participating naprapaths may have a relatively narrow view of EBP, or possibly, that this feature of EBP has not been well communicated among Swedish naprapaths. It may be that Swedish naprapathic respondents overestimated their skills and knowledge in EBP and, while being positive about EBP, are not fully aware of the complete dimensions of EBP [38]. As naprapathy can be considered to be on the margins of public-funded health care and higher education, and the majority of respondents had been in practice for eleven or more years, many naprapathic providers may not have received, or been exposed to, quality or recent training in EBP. Clear communication and education around patient preferences, alongside other principal EBP features as well as studies on naprapathy from the patient perspective could represent future key areas of professional development and research for the naprapathic profession.

### Skills of EBP

Participating naprapaths typically reported moderate to moderate-high skill levels for most EBP related tasks. The highest skill levels were in the task areas of problem identification (identifying answerable clinical questions and knowledge gaps in practice), evidence acquisition (locating professional literature and database searching online), and evidence application (applying research evidence to patient cases). In contrast, the lowest skill levels related to evidence generation (conducting systematic reviews and clinical research). Similar skill levels have been reported among other manual therapy professions, in Sweden [13, 34], Europe [14, 15, 17], Australia [16], and USA [4]. While manual therapists in clinical practice may not be required to possess high-level skills in generating or conducting clinical research, there is an expectation that they have sufficient skills in identifying, retrieving, assessing and applying research findings to clinical practice considering the importance of providing best practice care to patients at the point of care [39, 40]. Thus, the relatively high levels of perceived skills reported in these areas are encouraging. Further, little is known about how naprapaths communicate knowledge and disseminate evidence within their community, for example by oral means outside of the scholarly literature, which may be a relevant focus for future qualitative investigation.

### Implementation of EBP

Despite favourable attitudes towards EBP and the perceived moderate level of EBP skill, the majority of respondents had engaged in the implementation of EBP activities infrequently in the previous month. These activities include basic tasks associated with EBP, such as using an online database to search for practice-related literature, reading clinical research and using these findings to assist clinical decision-making. There appears to be a discord between the respondents’ attitudes towards EBP and the degree to which EBP is implemented or influences their clinical practice approach. This discord may not be unique to naprapaths as similar findings have been identified amongst other professions and jurisdictions [4, 13–16, 34]. These findings warrant further examination in future studies investigating EBP implementation in naprapathy and other health professions.

Encouragingly, the majority of respondents reported using clinical practice guidelines to a moderate extent to inform their clinical decision-making. However, it may be of some concern that traditional knowledge was the information source that most naprapaths used a lot or always to inform their clinical decision-making. Traditional knowledge may be defined as “knowledge, know-how, skills and practices that are developed, sustained and passed on from generation to generation within a community” [41], such as within the naprapathy community context. Notably, the use of traditional knowledge as an important source of information for clinical decision-making is consistent with findings of previous EBP studies examining EBP in manual therapy professions [13, 14, 16, 17, 34].

Naprapathy is a relatively small profession, mainly practised in the Nordic counties and the USA, accordingly there is a scarcity of literature on this profession. Furthermore, there is a general lack of inclusion of naprapathy in clinical research and clinical practice guidelines, vis-à-vis the more common inclusion and investigation of isolated manual therapy techniques such as massage or joint mobilisation/manipulation. The use of traditional knowledge as a leading clinical information source for naprapaths may in all likelihood be a product of this environment.

The prevailing use of traditional knowledge in naprapathic decision-making also may reflect the long-term marginalization of the naprapathic profession. In part, this may stem from the profession's reliance on educational and practice contexts outside of public funded and research-based educational and health care institutions. It is possible that the proportions of naprapaths using clinical practice guidelines versus traditional knowledge to inform their clinical decision-making may change once more research in naprapathy is published and implemented. Over time, this will help enhance the research culture, and contribute to the development of resources, aimed specifically at improving EBP implementation in the naprapathy profession. However, for this to happen, it is important that opportunities are made available to build research capacity within the profession, such as the development of authoritative educational advancements that enable naprapaths to undertake post-graduate academic studies.

### Training in EBP

The majority of respondents reported some degree of training related to EBP, although the level of training is unknown with about one third indicating they had received this during their undergraduate training programme. This mirrors the reported level of EBP training among Australian and UK osteopaths, which was predominately a minor part of an osteopathic educational programme [16, 17]. More than one-third of naprapathy respondents had undertaken no training in conducting clinical research or conducting systematic reviews or meta-analyses, a finding echoed in other studies investigating EBP in manual therapy professions [4, 16, 17]. It may be that a lack of exposure to adequate and coherent training in EBP has resulted in a relatively low level of implementation of EBP in naprapathic practice, despite favourable attitudes to EBP. Further professional development of EBP skills through undergraduate and postgraduate programmes and continuing education, and studies thereof, appears warranted. This may include investigations into the impact of EBP training on student and/or clinician understanding, self-efficacy and application of EBP.

### Barriers and enablers of EBP

Few responding naprapaths perceived major barriers to EBP uptake among those listed in the survey. Neither did naprapaths perceive a lack of clinical evidence as a major or moderate barrier to EBP uptake, as has been reported by osteopaths both in Australia and Europe [15–17, 34]. By contrast, most naprapaths indicated that each of the potential enablers of EBP uptake would be moderately to very useful. Factors reported as very useful enablers of EBP uptake were access to the internet and free online databases in the workplace, which is in line with findings of other manual therapy professions [13–17, 34].

Considering that access to the internet and free online databases in the workplace have repeatedly been reported as beneficial enablers of EBP uptake among manual therapy professions, and the now ubiquitous role of the internet in professional life alongside the availability of an increasing number of open access journals and online medical databases (such as PubMed, PEDro and The Cochrane Library), the conformingly low implementation of EBP activities in clinical practice among naprapaths and other manual therapy professions is somewhat puzzling. Arguably, there remain many research journals that are not available without subscription or academic affiliation, which may hinder clinician uptake of EBP. An important focus for future research would be to ascertain whether improved access to the internet, online databases and open-access publications (e.g. publications/databases provided through membership of professional associations), would provide an impetus for improving EBP implementation in naprapathy (as evidenced by higher EBP use scores).

### Methodological considerations

There are some limitations to the current study. The self-reporting survey design suggests the study may be susceptible to recall bias (e.g. overreporting of certain behaviours such as EBP activities), cognitive bias (e.g. confirmation or self-serving biases when answering survey questions), and/or self-selection bias (e.g. a potentially greater willingness to participate among respondents that were positive towards EBP). Although the English version of EBASE has been psychometrically evaluated and has demonstrated good content validity, construct validity and internal consistency, as well as acceptable test–retest reliability [25, 26], the Swedish version has not. Notwithstanding, the translation of the Swedish version of EBASE did adapt the WHO process for translating instruments [31], was pilot tested, utilised a secure web based survey hosting system widely used in Swedish higher education [32], and was conducted anonymously. These features can be considered strengths of the current study. Lastly, future survey studies might want to consider providing some ethically sound incentives to increase participation rate, e.g. continuing education credits or a small gift like a coffee voucher upon survey completion.

## Conclusions

This is the first study to investigate the attitudes, skills and implementation of EBP among licensed naprapaths in Sweden. The naprapaths that participated in this survey reported positive attitudes toward EBP and moderate levels of EBP skills, whereas the implementation of EBP was infrequent. The findings may inform the design and key areas for further EBP studies and professional development for the naprapathic profession.

## Data Availability

The data analysed during the current study, in aggregated format to maintain anonymity, are available from the corresponding author on reasonable request.
